# An Information Quantity in Pure State Models

**DOI:** 10.3390/e24040541

**Published:** 2022-04-12

**Authors:** Fuyuhiko Tanaka

**Affiliations:** 1 Graduate School of Engineering Science, Osaka University, Osaka 560-8531, Japan; ftanaka@sigmath.es.osaka-u.ac.jp; 2 Center for Quantum Information and Quantum Biology, Osaka University, Osaka 560-8531, Japan

**Keywords:** minimum entropy, facility location problem, quantum information, complex projective space, 81P17, 81P05, 81P45, 90B99, 53A20

## Abstract

When we consider an error model in a quantum computing system, we assume a parametric model where a prepared qubit belongs. Keeping this in mind, we focus on the evaluation of the amount of information we obtain when we know the system belongs to the model within the parameter range. Excluding classical fluctuations, uncertainty still remains in the system. We propose an information quantity called purely quantum information to evaluate this and give it an operational meaning. For the qubit case, it is relevant to the facility location problem on the unit sphere, which is well known in operations research. For general cases, we extend this to the facility location problem in complex projective spaces. Purely quantum information reflects the uncertainty of a quantum system and is related to the minimum entropy rather than the von Neumann entropy.

## 1. Introduction

Building a large-scale quantum computer remains challenging, and there are many problems to be solved. For example, the performance of error correction depends very strongly on how coherent the noise process is [1], and experimenters need to improve the quantum computing system through analysis of the physical noise [2]. When we prepare an imperfect quantum computing system, it is important to specify the noise based on a suitable error model. The process of understanding the physical error model for a prepared system corresponds to that of obtaining a certain amount of information on the system. In the present paper, keeping this in mind, we consider how to evaluate such information *without any entropic concept*.

An overly simple example is given by a purely rotation error model [2] with a parameter range. Let the ideal qubit state be |0〉 and the error model be $e^{-i\theta Y}|0\rangle$, where Y=−i(|0〉〈1|−|1〉〈0|) and θ denotes the parameter to be specified. Then the parameter range reflects the information we have on the system. We have more information when we know −ϵ≤θ≤ϵ than when we know −2ϵ≤θ≤2ϵ.

However, what if we consider a more complicated situation such as $|\theta_{1},\theta_{2}\rangle$ = $e^{-i\theta_{2}Z}$$e^{-i\theta_{1}Y}|0\rangle$, where Z=|0〉〈0|−|1〉〈1|, with the parameter range of, say, $0\leq \theta_{1}\leq \pi /4$ and $0\leq \theta_{2}\leq \pi /4$? How do we compare the range with the parameter range $0\leq \theta_{1}\leq 2\pi /5$ and $\theta_{2}=0$? To simplify the problem, let us adopt discrete models. We consider two situations: First, the qubit is described by one of the candidate pure states, |0〉,|+〉,|Y+〉, where $\left|+\rangle =\right|\theta_{1}=\pi /4,\theta_{2}=0\rangle$ and $\left|Y+\rangle =\right|\theta_{1}=\pi /4,\theta_{2}=\pi /4\rangle$. Second, it is described by one of the candidate pure states $|0\rangle ,\frac{|0\rangle +\sqrt{2}|1\rangle }{\sqrt{3}},|+\rangle$. Then, which state’s information is greater? Or equivalently, which uncertainty is larger? We will give a definite answer in the present article (See Section 8).

Let us describe our problem in a slightly more formal way before going into detail. Let the quantum system be described in a separable Hilbert space and a subset of pure states $M=\{\psi_{1},\psi_{2},\ldots \}$ be given. We call the subset M a *(pure-state) model*. Suppose we know that the quantum system is described by one of the pure states in the model. Then we evaluate the amount of information obtained by knowing the model.

We write the model as a countable set for simplicity, but a model might consist of an uncountable set of pure states, e.g., the above parametric pure state $|\theta_{1},\theta_{2}\rangle$. Another continuous model would be a wavefunction with a certain continuous parameter {φ(x−s): −∞<s<∞} (see, e.g., Holevo [3]).

Our problem is closely related to so-called quantum estimation, but the above type of problem has still not been investigated. In quantum state estimation [4,5,6,7,8,9] or quantum state discrimination [6,10,11,12,13], for a given model, we find an optimal quantum measurement to extract information on the quantum state and choose the true state in the model from observation. This has been a typical problem and has been investigated by many authors. In quantum information experiments, quantum tomography has been also discussed [14,15,16,17]. As far as the author knows, these studies do not refer to the comparison of several models in terms of information quantity.

In our setting, we focus on the information that we obtain *before* preparation for a measurement. As we see later, we clearly obtain a certain amount of information other than the dimension of the Hilbert space.

We consider only pure state models so that we can neglect classical fluctuation. As we shall see later, there is no classical counterpart for such information. In other words, we calibrate so that such classical information becomes zero. If a positive amount of information remains under the calibration, then we expect that it reflects the truly quantum information. We do not have a proper name for this information, and we call it *model information* or *information of the model* tentatively. It would become an alternative to the usual entropy.

In the next section, we provide a rough idea of how to define model information and present pure state models as examples. Then, we will formulate a pure state model and define the representative quantum state for it in a rigorous manner. In Section 4, we describe the equivalence between the problem of finding the representative quantum state and finding the minimax facility location on the sphere in operations research. In Section 5, we introduce the purely quantum information of the model and calculate it in several examples. We also describe the relationship of entropic concepts to our result and extension to infinite-dimensional Hilbert space in Section 7. Finally, concluding remarks are given in Section 8.

## 2. Rough Idea on Defining Model Information

### 2.1. Preliminary Considerations

In this section, we describe a rough idea of how we evaluate model information. First, we recall classical information theory. Suppose that Alice picks a three-letter word $w_{1}w_{2}w_{3}$, where $w_{1},w_{2},w_{3}\in \left\{a,b,c,\ldots ,z\right\}$ and we set M={a,b,c,…,y}. If Bob knows $w_{1}\in M$, Bob does not feel that he obtains much information. However, if $w_{1}\in M^{\prime }=\left\{a,e,i,o,u\right\}$, then Bob feels that he obtains more information on the word Alice picks.

The above situation corresponds to a commutative case in quantum theory. Keeping this in mind, let us consider the model information in the quantum system. We assume that Bob already knows that the quantum system is described in a *d*-dimensional Hilbert space. Since information quantity is a relative concept, let us compare two models. Let the first model consist of a *d*-dimensional orthonormal basis, i.e., $M=\{e_{1},\ldots ,e_{d}\}$ and the second model consist of $\left\{e_{1},\ldots ,e_{k}\right\}$ (k<<d). At least we can say that the second model gives more significant information to Bob than the first model. This is because the quantum state is in a proper subspace.

Now we tackle the case where some quantum states are nonorthogonal. For simplicity, we set d=2 and consider the following models:

$$\begin{array}{cc} M_{1} & =\{|+\rangle ,|-\rangle \},\\ M_{2} & =\{|0\rangle ,|+\rangle \},\\ M_{3} & =\{|0\rangle ,\frac{|0\rangle +\sqrt{2}|1\rangle }{\sqrt{3}},|+\rangle \},\\ M_{4} & =\{|0\rangle ,|+\rangle ,|Y+\rangle \}, \end{array}$$

where $|\pm \rangle =\frac{1}{\sqrt{2}}(|0\rangle \pm \left|1\rangle \right)$, $|Y+\rangle =\frac{1}{\sqrt{2}}(|0\rangle +i|1\rangle )$. In explicit calculations, we set $|0\rangle ={\left(1,0\right)}^{⊤},|1\rangle ={\left(0,1\right)}^{⊤}$.

Suppose that we know that the quantum state φ is one of the candidate states in $M_{2}$ (hereinafter, we write $\varphi \in M_{2}$ for simplicity). Perhaps we agree that the information is more than $\varphi \in M_{3}$ and $\varphi \in M_{4}$. Then, which is more informative, $\varphi \in M_{3}$ or $\varphi \in M_{4}$? Both models consist of three nonorthogonal state vectors. Likewise, which is more informative, $\varphi \in M_{1}$ or $\varphi \in M_{2}$? In the present article, we consider how to quantitatively evaluate the information obtained when Bob knows that the quantum state φ belongs to a model M.

### 2.2. Full Rank Condition

In order to avoid technical difficulties, we give one important assumption here. Let us define the *rank* of a model as

$$\begin{array}{c} \mathrm{rank}M=\dim{\mathrm{span}}_{C}\left\{\psi_{1},\psi_{2},\ldots \right\}. \end{array}$$

We assume that the rank of a model is equal to the dimension of the Hilbert space, i.e., d=rankM. We call it the *full rank condition*. The full rank condition implies that there exists no complementary subspace that is orthogonal to every state vector in the model M.

### 2.3. Rough Idea on Defining Model Information

Under the full rank condition, we consider the case where a model has considerable information on the quantum system. Suppose that we are given the following model:

$$\begin{array}{c} M=\{|e_{1}\rangle ,\sqrt{1-\epsilon }|e_{1}\rangle +\sqrt{\epsilon }|e_{2}\rangle ,\ldots ,\sqrt{1-\epsilon }|e_{1}\rangle +\sqrt{\epsilon }|e_{d}\rangle \} \end{array}$$

(0<ϵ<<1). While this satisfies the full rank condition, clearly all candidate quantum states are approximately in the same direction as $|e_{1}\rangle$.

Then, the quantum system φ is approximately described by a representative state vector $|e_{1}\rangle$. When ϵ→0 but ϵ≠0, the model information is expected to increase.

From the above discussion, we find that the information quantity associated with φ∈M is completely different from the number of elements, |M|. Rather, a certain scale or a size of the model M should be included in the definition of the model information.

Along the lines of the above rough idea, we discuss in the next section:

(a) How to determine a representative state vector for a given model M;
(b) One definition of the model information;
(c) The relationship with the concept of entropy.

We emphasize that all of these have no classical counterpart and thus it might be difficult to understand them. Before going into detail, we shall give an overview of each item here.

For (a), we consider maximin overlap between quantum states and define the representative quantum state of a model. Mathematically speaking, it is regarded as a variant of the facility location problem on the unit sphere [18,19], which appears in operations research. In operations research, many authors have developed algorithms on the facility location problem. In particular, finding the minimax solution is our concern. For a finite model (|M|<∞), we present a naive algorithm to find the representative quantum state for a model using this consideration.

In order to consider item (b), we introduce an imaginary two-person game called the quantum detection game. Bob benefits from the information of a given model to obtain a higher score than Alice. The value of the game, which is determined by a least favorable prior [20] in this game, defines one information quantity related to the model M.

In (c), we compare our method with the formal analogue based on the von Neumann entropy. Later we will see that the newly proposed information quantity is related to the minimum entropy [21,22] rather than the von Neumann entropy.

## 3. Basic Definitions

### 3.1. Definition of Pure State Models and Assumptions

In the present paper, let H be a *d*-dimensional Hilbert space. (*d* could be *∞*). We call a finite-dimensional parametric family of quantum pure states

$$\begin{array}{c} M=\{\psi_{\theta }\in H\;:\;∥\psi_{\theta }∥=1,\theta \in \Theta \subset R^{m}\} \end{array}$$

a *quantum statistical model of pure states* or briefly a *(pure state) model*. Note that ${∥f∥}^{2}=\langle f|f\rangle$. Basically, the parameter set Θ is a compact subset of finite-dimensional Euclidean space.

We assume the following two conditions:

(1) Identifiability: $\theta \neq \theta^{\prime }\Rightarrow |\psi_{\theta }\rangle \langle \psi_{\theta }\left|\neq \right|\psi_{\theta^{\prime }}\rangle \langle \psi_{\theta^{\prime }}|$. Conventionally, we only consider quantum states up to the (global) phase factor below, and we often identify a pure state |ψ〉 with a density operator |ψ〉〈ψ|.
(2) Continuity: For every sequence ${\left\{\theta_{n}\right\}}_{n=1}^{\infty }\subset \Theta$ and θ∈Θ,
  $$\begin{array}{c} \theta_{n}\to \theta \Rightarrow {∥|\psi_{\theta_{n}}\rangle \langle \psi_{\theta_{n}}\left|-\right|\psi_{\theta }\rangle \langle \psi_{\theta }|∥}_{\infty }\to 0 \end{array}$$
  holds. (${∥A∥}_{\infty }$ denotes the operator norm, i.e., ${∥A∥}_{\infty }=\sup_{∥\varphi ∥=1}∥A\varphi ∥$).

For simplicity, we often consider a finite set of parameters, $\Theta =\{\theta_{1},\ldots ,\theta_{k}\}$. Then, $\psi_{\theta_{j}}$ is denoted by $\psi_{j}$. We often call it a *discrete model*, which is written as $M=\{\psi_{1},\ldots ,\psi_{k}\}$.

### 3.2. Preliminary Results

In the present paper, we introduce the information of a model M. Although the formal definition is given in Section 5, we need several concepts to understand them analytically and geometrically.

In this section, we introduce the most fundamental concept, the representative quantum state of a model. We shall give a rough idea for when dimH=2 and |M|=2. Specifically, we set $M=\{\psi_{1},\psi_{2}\}$ with $∥\psi_{1}∥=∥\psi_{2}∥=1$. When two quantum states are close to each other, $\psi_{1}≃\psi_{2}$, it is natural to consider that a representative quantum state of model M should be a “midpoint between two quantum states”. We often identify the state vector with the point on the whole pure states specified by the vector.

Mathematically, we may try to define the point as the point φ equidistant between $\psi_{1}$ and $\psi_{2}$ such that

(1) $$\begin{array}{c} |\langle \varphi |\psi_{1}\rangle \left|=\right|\langle \varphi |\psi_{2}\rangle | \end{array}$$

holds.

However, the above equidistance condition does not determine the point φ generally. Thus, we maximize the above “overlap” under the condition (1). Then we obtain an explicit formula for the representative point of a model,

(2) $$\begin{array}{c} |\varphi_{rep}\rangle =\frac{|\psi_{1}\rangle +e^{-i\delta }|\psi_{2}\rangle }{\sqrt{2(1+|\gamma |)}},\;\gamma =\langle \psi_{1}|\psi_{2}\rangle ,\delta =\arg\gamma \end{array}$$

where δ satisfies $\gamma =\left|\gamma \right|e^{i\delta }$ and then the maximum overlap is given by $\max_{\varphi }\left|\langle \varphi |\psi_{1}\rangle \right|=\frac{1+|\gamma |}{2}$.

Next, we consider the case where |M|=3. Let us take $M_{3}$ and $M_{4}$ introduced in the previous section:

In $M_{4}$, the above idea applies, i.e., we find the quantum state to maximize the overlap

(3) $$\begin{array}{c} |\langle \varphi |\psi_{1}\rangle \left|=\right|\langle \varphi |\psi_{2}\rangle \left|=\right|\langle \varphi |\psi_{3}\rangle |. \end{array}$$

Up to the global phase, we set φ as

(4) $$\begin{array}{c} |\varphi \rangle =\left(\begin{array}{c} \cos\alpha \\ \sin\alpha \;e^{i\delta } \end{array}\right),\;0\leq \alpha \leq \pi /2,\;0\leq \delta <2\pi . \end{array}$$

Then, we obtain an explicit solution satisfying (3), $\cos\alpha =\sqrt{\frac{3+\sqrt{3}}{6}},\delta =\frac{\pi }{4}$ after lengthy but straightforward algebra. (See also Section 4.1.3).

However, we find no solution satisfying Equation (3) in $M_{3}$. We need a more careful treatment. First, we fix an arbitrary quantum state φ and consider the set of numbers *r* satisfying $|\langle \varphi |\psi_{j}\rangle |\geq r$, j=1,2,3. The condition assures that the overlap between φ and an arbitrary quantum state in M is not less than *r*. For each φ, the maximum of *r* is equal to $\min\{|\langle \varphi |\psi_{j}\rangle |:\;j=1,2,3\}$.

We consider that the larger the overlap gets, the more suitable φ becomes as a representative quantum state of the model M. Thus, we maximize *r* as a function of φ.

It is convenient for explicit calculation to use the squared overlap (i.e., Fidelity), $\max_{\varphi }\min_{\psi \in M_{3}}{\left|\langle \varphi |\psi \rangle \right|}^{2}$, and we regard $\varphi_{rep}=\arg\max r\left(\varphi ;M_{3}\right)$ as a representative quantum state of the model $M_{3}$. Based on the above idea, we will give a more formal definition of the representative quantum state in the next subsection.

### 3.3. Representative Quantum State

Now we are ready to define the representative quantum state of a given model M formally. We adopt the distance $d_{F}\left(\varphi ,\varphi^{\prime }\right)=1-F\left(\varphi ,\varphi^{\prime }\right)=1-{\left|\langle \varphi |\varphi^{\prime }\rangle \right|}^{2}$ rather than the overlap.

**Definition** **1.** *Let a model M be given. When a quantum state $\varphi_{rep}$ satisfies*

(5) $$\begin{array}{cc} \underset{\varphi }{\inf}\underset{\psi \in M}{\sup}d_{F}\left(\varphi ,\psi \right) & =\underset{\psi \in M}{\sup}d_{F}\left(\varphi_{rep},\psi \right), \end{array}$$

*$\varphi_{rep}$ is called a representative quantum state of the model M with respect to the distance $d_{F}$.*

When we emphasize the model M, we write $\varphi_{rep}\left(M\right)$. While the terms max and min are enough for discrete models, using the terms sup and inf is generally inevitable. (see Section 7). We also use a condition equivalent to (5),

$$\begin{array}{cc} \underset{\psi \in M}{\sup}d_{F}\left(\varphi ,\psi \right) & \geq \underset{\psi \in M}{\sup}d_{F}\left(\varphi_{rep},\psi \right),\;\forall \varphi . \end{array}$$

In the above definition, $\varphi_{rep}$ is also interpreted as the minimax estimate in quantum estimation with *no observation*. Suppose that a parametric family of pure states or countable set of pure states is given. Then we give an estimate, say φ, as the true quantum state without any observation. The error is evaluated by the Fidelity-based quantity, $d_{F}\left(\varphi ,\psi_{true}\right)=1-{\left|\langle \varphi |\psi_{true}\rangle \right|}^{2}$. The above representative quantum state is a minimax estimate in this setting.

In the context of quantum estimation, this may seem quite strange because we do not perform any measurement. However, it is not unnatural to consider estimation with no observation. For example, in classical information theory, we infer the outcome of an information source with no observation. For a given parametric model of source code distribution $\left\{p_{\theta }\left(x\right):\theta \in \Theta \right\}$, this kind of estimation corresponds to constructing a minimax code [23].

Apart from actual application, quantum estimation with no observation also makes sense theoretically. In a quantum computer, a quantum bit will be processed under a certain quantum gate with an unknown parameter, say, θ, during the computing process. When θ is uncontrollable with a range [−2ϵ,ϵ], it might be necessary to estimate the quantum bit. Since there is no reason to estimate θ≃0.5ϵ, we need a certain formulation to estimate the quantum bit.

We should also mention why we adopt $d_{F}$ as the distance among several candidates as the closeness measure in our definition. There are two reasons. One is the operational meaning of the quantum detection game, which is explained in Section 5. The other is due to the following property:

**Lemma** **1.** *Let a model $M=\{\psi_{\theta }\}$ be given. Let f be a continuous nondecreasing function on [0,1]. When we adopt the distance $d\left(\varphi ,\psi \right)=f\circ d_{F}\left(\varphi ,\psi \right)$, then the representative quantum state remains the same.*

**Proof.**  It is enough to show that for every φ,

(6) $$\begin{array}{cc} f\left\{\underset{\theta }{\sup}d_{F}\left(\varphi ,\psi_{\theta }\right)\right\} & =\underset{\theta }{\sup}f\circ d_{F}\left(\varphi ,\psi_{\theta }\right) \end{array}$$

holds.

If Equation (6) holds true, then we show the statement in the following way. For every φ, from the definition of $\varphi_{rep}$, $\sup_{\theta }d_{F}\left(\varphi ,\psi_{\theta }\right)\geq \sup_{\theta }d_{F}\left(\varphi_{rep},\psi_{\theta }\right)$ holds. Since *f* is an increasing function, applying *f* to both sides and using Equation (6) yields $\sup_{\theta }f\circ d_{F}\left(\varphi ,\psi_{\theta }\right)$$\geq \sup_{\theta }f\circ d_{F}\left(\varphi_{rep},\psi_{\theta }\right)$, which implies that $\varphi_{rep}$ is a representative quantum state with respect to the distance $f\circ d_{F}$.

Now let us show Equation (6). Let φ be fixed and set $\alpha =\sup_{\theta }d_{F}\left(\varphi ,\psi_{\theta }\right)$. For every ϵ>0, due to the continuity of *f*, there exists δ>0 such that |α−x|≤δ⇒f(α)≤f(x)+ϵ. We take $\theta_{*}$ such that $\alpha \leq d_{F}\left(\varphi ,\psi_{\theta_{*}}\right)+\delta$. Then

$$\begin{array}{cc} f(\alpha ) & \leq f(d_{F}\left(\varphi ,\psi_{\theta_{*}}\right))+\epsilon \\ & \leq \underset{\theta }{\sup}f\circ d_{F}\left(\varphi ,\psi_{\theta }\right)+\epsilon \end{array}$$

Since ϵ is arbitrary, we obtain $f\left(\alpha \right)\leq \sup_{\theta }f\circ d_{F}\left(\varphi ,\psi_{\theta }\right)$.

Next, observe that $f\left(\alpha \right)\geq f\circ d_{F}\left(\varphi ,\psi_{\theta }\right)$ for every θ since $\alpha \geq d_{F}\left(\varphi ,\psi_{\theta }\right)$. Taking the supremum of RHS with respect to θ, we obtain the converse inequality. Thus, Equation (6) is shown, and the proof is complete. □

In Section 5, we shall define the information quantity obtained when we know φ∈M, which is denoted by J(M). When we find $\varphi_{rep}$, it is shown to be easy to calculate J(M).

Now let us consider the representative quantum state of a two-state model geometrically. Recall that each pure state in a two-dimensional Hilbert space is written in the form (4). If we switch to the Bloch representation, we obtain one-to-one correspondence between (α,δ)↔(x,y,z)=(sin2αcosδ,sin2αsinδ,cos2α) on the unit sphere (Bloch sphere). When one pure state is set to |0〉 (*P*), the distance between the pure state and another pure state specified with (α,δ) (*Q*) is 2α along the shortest path on the Bloch sphere. The shortest path connecting two points *P* and *Q* on the Bloch sphere is the arc along the large circle on the Bloch sphere. The arc is called a *geodesic* connecting *P* and *Q* and the equidistant point *M* on the geodesic from both points is called the *geodesic midpoint between P and Q*. The representative quantum state corresponds to the geodesic midpoint. The concept of geodesics on the Bloch sphere is often useful and has been investigated in several works [24,25,26,27].

For every pair of independent quantum states $\psi_{1}$ and $\psi_{2}$, let us consider a two-dimensional subspace ${\mathrm{span}}_{C}\left\{\psi_{1},\psi_{2}\right\}$. Then each state in the subspace is regarded as a point on the Bloch sphere. By using the Formula (2), we summarize the above statements.

**Lemma** **2.** *Let a model M be given. Then, for every pair of quantum states $\psi_{1}$ and $\psi_{2}$ with $∥\psi_{1}∥=∥\psi_{2}∥=1$, the geodesic midpoint $\varphi_{M}$ is given by*

(7) $$\begin{array}{c} |\varphi_{M}\rangle =\frac{|\psi_{1}\rangle +e^{-i\delta }|\psi_{2}\rangle }{\sqrt{2(1+|\gamma |)}},\;\gamma =\langle \psi_{1}|\psi_{2}\rangle ,\;\delta =\arg\gamma \end{array}$$

*where δ satisfies $\gamma =\left|\gamma \right|e^{i\delta }$ and then arc length α between $\varphi_{M}$ and $\psi_{1}$ is given by $\alpha =\arccos\left|\langle \psi_{1}|\psi_{2}\rangle \right|$ and arc length between $\psi_{1}$ and $\psi_{2}$ is 2α.*

Understanding the geometry of the unit sphere is very helpful to find the representative quantum state, which is discussed in Section 4.

### 3.4. Example of a Representative Quantum State: $M_{3}$

As a slightly nontrivial example, let us focus on $M_{3}$ and find a representative quantum state. First, we focus on the submodel $N_{3}=\{|0\rangle ,\frac{|0\rangle +\sqrt{2}|1\rangle }{\sqrt{3}}\}$. Then its representative quantum state, $\varphi_{rep}\left(N_{3}\right)$ is the geodesic midpoint of |0〉 and $\frac{|0\rangle +\sqrt{2}|1\rangle }{\sqrt{3}}$. Using the Formula (7),

(8) $$\begin{array}{cc} |\varphi_{rep}\left(N_{3}\right)\rangle & =\frac{\left(\sqrt{3}+1\right)|0\rangle +\sqrt{2}|1\rangle }{2\sqrt{\left(\sqrt{3}+1\right)}} \end{array}$$

is obtained.

Next, we use the following lemma.

**Lemma** **3.** *Let a model M and its submodel N⊂M be given. If the representative quantum state $\varphi_{rep}$ of N satisfies*

$$\begin{array}{cc} \underset{\psi \in N}{\sup}d_{F}\left(\varphi_{rep},\psi \right) & =\underset{\psi \in M}{\sup}d_{F}\left(\varphi_{rep},\psi \right), \end{array}$$

*then $\varphi_{rep}$ is also the representative quantum state of M.*

**Proof.**  Let φ be an arbitrary quantum state. Since N⊂M,

$$\begin{array}{cc} \underset{\psi \in M}{\sup}d_{F}\left(\varphi ,\psi \right) & \geq \underset{\psi \in N}{\sup}d_{F}\left(\varphi ,\psi \right)\\ & \geq \underset{\psi \in N}{\sup}d_{F}\left(\varphi_{rep},\psi \right)\\ & =\underset{\psi \in M}{\sup}d_{F}\left(\varphi_{rep},\psi \right), \end{array}$$

which implies that $\varphi_{rep}$ is also the representative quantum state of M. □

It is easily seen that $d_{F}(\varphi_{rep}\left(N_{3}\right),|+\rangle )<\max_{\psi \in N_{3}}d_{F}\left(\varphi_{rep}\left(N_{3}\right),\psi \right)$. Due to the above Lemma 3, $\varphi_{rep}\left(N_{3}\right)$ (8) is also the representative quantum state of $M_{3}$.

## 4. Facility Location Problem

Mathematically, finding the representative quantum state of a given model is equivalent to finding the minimax facility location for a given demand point in operations research [18].

### 4.1. Facility Location Problem on the Sphere

Decades ago, Drezner and Wesolowsky [18] considered the facility location problem on the sphere. We briefly summarize their formulation. Suppose that there are *m* demand points with (positive) weights on the unit sphere and our objective is to locate a single facility on the same sphere so as to minimize the weighted sum of distances from the facility to the demand points. Let $\left(\xi_{i},\phi_{i}\right)$ and (ξ,ϕ) denote the locations of the *i*-th demand point and the facility, respectively, in spherical coordinate (0≤ξ≤π, 0≤ϕ<2π). Weights for demand points are denoted as $\pi_{i}$. Without loss of generality, we may take $\sum_{i}\pi_{i}=1$. We obtain the following minimization problem.

$$\min_{\xi ,\phi }\sum_{i=1}^{m}\pi_{i}d_{i}\left(\xi ,\phi \right),$$

where $d_{i}\left(\xi ,\phi \right)$ is the distance between the facility (ξ,ϕ) and the *i*-th demand point $\left(\xi_{i},\phi_{i}\right)$.

Drezner and Wesolowsky measured distances through the sphere for squared Euclidean distances and they also used the shortest length of arc. Let us denote the shortest length of arc between two points on a sphere with a unit radius by α. Then the squared Euclidean distance is given by $4\sin^{2}\left(\alpha /2\right)$. Both distances are computed by the equation:

$$\begin{array}{c} \cos\alpha =\cos\xi_{i}\cos\xi +\sin\xi_{i}\sin\xi \cos\left(\phi_{i}-\phi \right). \end{array}$$

The interpretation of the problem is as follows. The distance $d_{i}\left(\xi ,\phi \right)$ is the transportation cost from a facility (ξ,ϕ) to the *i*-th demand point $\left(\xi_{i},\phi_{i}\right)$. We regard the relative frequency of each transport as the weight. When we already know the relative frequency, we minimize the objective function $\sum_{i=1}^{m}\pi_{i}d_{i}\left(\xi ,\phi \right)$ with respect to (ξ,ϕ).

Focusing on the correspondence between a point (ξ,ϕ) on the unit sphere and a complex unit vector

(9) $$\begin{array}{cc} |\psi (\xi ,\phi )\rangle & =\left(\begin{array}{c} \cos\xi /2\\ e^{i\phi }\sin\xi /2 \end{array}\right), \end{array}$$

the problem is completely solved when we adopt the squared Euclidean distance. Let us denote |φ〉=|ψ(ξ,ϕ)〉 as the location of the facility instead of (ξ,ϕ). Straightforward calculation yields $d_{i}\left(\xi ,\phi \right)=4\left\{1-{\left|\langle \varphi |\psi \left(\xi_{i},\phi_{i}\right)\rangle \right|}^{2}\right\}=4d_{F}\left(\varphi ,\psi \left(\xi_{i},\phi_{i}\right)\right)$. Thus, the objective function to be minimized is written as

$$\begin{array}{cc} \sum_{i=1}^{m}\pi_{i}d_{i}\left(\xi ,\phi \right) & =4\sum_{i=1}^{m}\pi_{i}\left\{1-{\left|\langle \varphi |\psi \left(\xi_{i},\phi_{i}\right)\rangle \right|}^{2}\right\}\\ & =4\langle \varphi |\left\{I-\left(\sum_{i=1}^{m}\pi_{i}|\psi \left(\xi_{i},\phi_{i}\right)\rangle \langle \psi \left(\xi_{i},\phi_{i}\right)|\right)\right\}|\varphi \rangle \\ & =4\left(1-\langle \varphi |\rho_{\pi }|\varphi \rangle \right), \end{array}$$

where in the last line, we set $\rho_{\pi }=\sum_{i=1}^{m}\pi_{i}|\psi \left(\xi_{i},\phi_{i}\right)\rangle \langle \psi \left(\xi_{i},\phi_{i}\right)|$. Note that $\rho_{\pi }$ is positive semidefinite and of trace one, and it is regarded as the *Bayes mixture* (For the definition, see Section 5.1). Then, the minimization problem $\min_{\varphi }\sum_{i=1}^{m}\pi_{i}d_{i}\left(\xi ,\phi \right)$ reduces to finding the maximum of $\langle \varphi |\rho_{\pi }|\varphi \rangle$. This is given by the first eigenvector φ of $\rho_{\pi }$. This result [28] agrees with that derived by Drezner and Wesolowsky, who obtained the same result by differentiation with respect to variables (ξ,ϕ) in the context of operations research.

However, what if we have no information on the relative frequency $\left\{\pi_{i}\right\}$ for each demand point? One idea is to take the minimax point. Through the minimax theorem [20], we obtain the associated weight $\pi^{*}=\left\{\pi_{i}^{*}\right\}$ such that

(10) $$\begin{array}{cc} \min_{\xi ,\phi }\sum_{i=1}^{m}\pi_{i}^{*}d_{i}\left(\xi ,\phi \right) & =\min_{\xi ,\phi }\max_{i=1,\ldots ,m}d_{i}\left(\xi ,\phi \right) \end{array}$$

holds, where $d_{i}\left(\xi ,\phi \right)$ is the squared Euclidean distance. (Strictly speaking, the above holds under the condition that the convex hull of all demand points does not include the origin). This $\pi^{*}$ is called the *least favorable weight*.

Now we go back to our problem. Let the Hilbert space be two-dimensional, where each quantum state is specified by (ξ,ϕ) in (9). Let a discrete model $M=\{|\psi \left(\xi_{1},\phi_{1}\right)\rangle ,\ldots$, $\left|\psi (\xi_{m},\phi_{m})\rangle \right\}$ be given. Each state corresponds to a demand point on the Bloch sphere. The distance $d_{F}$ corresponds to the transportation cost measured by a constant multiplied by the squared Euclidean distance. Then, finding the representative quantum state $\varphi_{rep}$ of the model M is equivalent to finding the minimax facility location specified by φ on the Bloch sphere with the distance $d_{F}$.

According to this correspondence, we also know the following fact. When we know the least favorable weight $\pi_{*}$ in the problem, we obtain the representative quantum state as the first eigenvector of the Bayes mixture $\rho_{\pi }$ with respect to $\pi_{*}$ [20].

Following the interpretation of the facility location problem on the sphere, we find the representative quantum state of each model in Section 2.1. Since $\sin^{2}\left(\alpha /2\right)$ is a strictly increasing function of α, both minimax points for the two distance measures agree due to Lemma 1.

#### 4.1.1. Example 1: $M_{2}$

**Figure 1:**
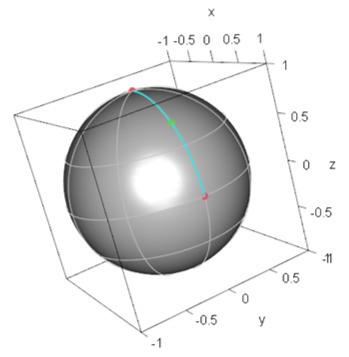
Configuration of demand points in $M_{2}$ and the minimax facility location on the Bloch sphere: The red solid points denote demand points and the green solid point denotes the minimax facility location (the representative quantum state), which is the geodesic midpoint.

Pure states |+〉 and |0〉 correspond to the demand points specified by (ξ,ϕ)=(π/2,0) and (ξ,ϕ)=(0,0) (North pole) on the Bloch sphere, respectively. Then, the minimax location on the Bloch sphere is specified by $\left(\xi_{M},\phi_{M}\right)=\left(\pi /4,0\right)$. Thus, $\varphi_{rep}$ of $M_{2}$ is given by $|\varphi_{rep}{\rangle =\left(\cos\pi /8,\sin\pi /8\right)}^{⊤}$. Figure 1 shows the demand points and the minimax location of the facility on the Bloch sphere.

#### 4.1.2. Example 2: $M_{3}$

**Figure 2:**
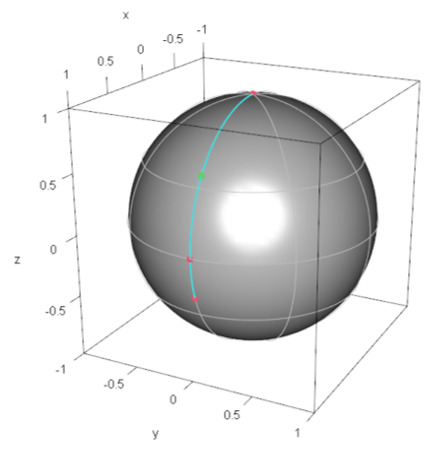
Configuration of demand points in $M_{3}$ and the minimax facility location on the Bloch sphere: The red solid points denote demand points and the green solid point denotes the minimax facility location (the representative quantum state), which is the geodesic midpoint between the most distant pair of demand points.

First, the quantum state $\frac{|0\rangle +\sqrt{2}|1\rangle }{\sqrt{3}}$ corresponds to the demand point specified by $\left(\xi_{*},0\right)$, where $\cos\xi_{*}/2=1/\sqrt{3},\sin\xi_{*}/2=\sqrt{2/3}$. We follow Algorithm Step (1) described in the next subsection. Then, the most distant pair is (ξ,ϕ)=(0,0) (North pole) and $\left(\xi_{*},0\right)$. The other point specified by (ξ,ϕ)=(π/2,0) is closer to the center specified by $\left(\xi_{*}/2,0\right)$ than that pair of points. Thus, the minimax location on the Bloch sphere is specified by $\left(\xi_{*}/2,0\right)$ and $\varphi_{rep}$ of $M_{3}$ is given by $|\varphi_{rep}\rangle ={\left(\cos\xi_{*}/4,\sin\xi_{*}/4\right)}^{⊤}$. Figure 2 shows the demand points and minimax location of the facility on the Bloch sphere.

#### 4.1.3. Example 3: $M_{4}$

**Figure 3:**
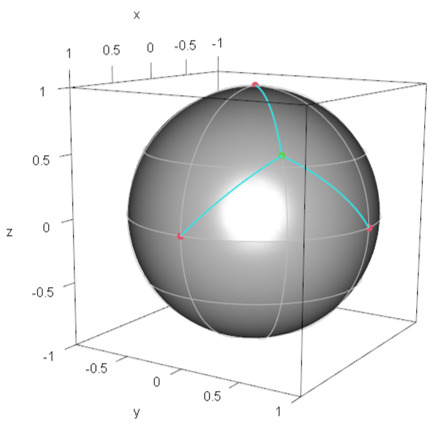
Configuration of demand points in $M_{4}$ and the minimax facility location on the Bloch sphere: The red solid points denote demand points, and the green solid point denotes the minimax facility location (the representative quantum state), which is the center of the circumscribed circle of the triangle whose edges are the demand points (See Algorithm Step (2)).

For $M_{4}$, we follow Algorithm Step (1) and Step (2) described in the next subsection. After tedious calculation, we find the minimax location, which is specified by $\left(\xi_{*}/2,\pi /4\right)$ ($\xi_{*}$ is defined as above) and $\varphi_{rep}$ of $M_{4}$ is given by $|\varphi_{rep}\rangle ={\left(\cos\xi_{*}/2,e^{i\frac{\pi }{4}}\sin\xi_{*}/2\right)}^{⊤}$. Figure 3 shows the demand points and the minimax location of the facility on the Bloch sphere.

It may be thought that using the squared Euclidean distance rather than the arc length is unnatural. However, as shown in Lemma 1, The minimax location obtained under the squared Euclidean distance agrees with that obtained under the arc length due to the strict monotonicity of $\alpha \mapsto 4\sin^{2}\left(\alpha /2\right)$ (See the beginning of Section 4.1 for the squared Euclidean distance). In this sense, *the representative quantum state of a model is invariant*. On the contrary, the least favorable weight for the facility location problem depends on the measure of distance on the sphere; thus, it is not invariant.

### 4.2. Algorithm to Find Nonrandomized Minimax Location

In operations research, there are several studies on the facility location problem on the unit sphere, where some algorithms to find the minimax facility location are also proposed. Inspired by these studies, we propose a naïve algorithm to find the nonrandomized minimax facility location. Specifically, we consider the facility location problem on a three-dimensional hypersphere in a four-dimensional *real* Euclidean space. This is easily generalized to a general dimension. Basically, a pure state model in a *d*-dimensional Hilbert space is regarded as a subset of a complex projective space $CP^{d-1}$. A complex projective space $CP^{d-1}$ is a typical example of a complex manifold but is actually a 2d−2-dimensional *real* manifold. This fact is sufficient to understand the following argument. (For complex projective space, e.g., see Section 4 in Bengtsson and Życzkowki [29]).

We exclude the possibility of a randomized strategy, although it is sometimes better than any nonrandomized strategy, at least theoretically. (For example, see Section 1.5 in Ferguson [30]). For example, let us consider six demand points, (ξ,ϕ)=(π/2±ϵ,0), (π/2±ϵ,2π/3), (π/2±ϵ,4π/3) on the unit sphere, where ϵ is a small positive constant. Then, a randomized facility location strategy, north pole with probability 1/2 and south pole with probability 1/2, yields the average transportation cost measured by the arc length, 1/2(π/2+ϵ)+1/2(π/2−ϵ)=π/2 for each demand point. Thus, it achieves the minimax location. On the other hand, any nonrandomized strategy yields a higher transportation cost (>π/2) in the worst case. The algorithm presented below fails in this example. When dimH=2, all demand points are not covered in a hemisphere if and only if there exists a randomized strategy that is better than any nonrandomized strategy. No simple mathematical condition can assure that nonrandomized minimax is not worse than any randomized strategy when dimH>2. Thus, we implicitly assume this fact and the existence of a nonrandomized minimax strategy in the Algorithm 1.
**Algorithm 1: Find Minimax Facility Location**(1)Find the most distant pair: Let two distinct demand points *A* and *B* be arbitrary. Then, from Formula (7), find the geodesic midpoint *P*. Then calculate the arc length AP (= BP). Find the maximum arc length $R_{2}$ and its center $P_{2,*}$. If every arc length between $P_{2,*}$ and a demand point is not more than $R_{2}$, then $P_{2,*}$ is the minimax location and STOP. If not, then go to Step (2).(2)Find the most distant triplet: Let *A*, *B*, and *C* be three arbitrary demand points. Find the center *P* of the circumscribed circle of the triangle ▵ABC on the hypersphere. Then calculate the arc length AP (= BP = CP). Find the maximum arc length $R_{3}$ (>$R_{2}$) and its center $P_{3,*}$. If every arc length between $P_{3,*}$ and a demand point is not more than $R_{3}$, then $P_{3,*}$ is the minimax location and STOP. If not, then go to Step (3).(3)Find the most distant quadruplet: Let *A*, *B*, *C*, and *D* be four arbitrary demand points. Find the center *P* of the circumscribed sphere of the tetrahedron ABCD on the hypersphere. Then, calculate the arc length AP (= BP = CP = DP). Find the maximum arc length $R_{4}$ (>$R_{3}$) and its center $P_{4,*}$. $P_{4,*}$ is the minimax location and STOP.

Due to monotonicity, we may evaluate the squared Euclidean distance or inner product instead of the arc length between two demand points.

When we generalize the algorithm suitably to $CP^{d-1}$ (as a 2d−2-dimensional real manifold), we obtain the algorithm to find the representative quantum state of a model in a *d*-dimensional Hilbert space. In the last subsection, we used the proposed algorithm to find the representative quantum state in some examples. In Section 5, we also demonstrate how to find the representative quantum state following the above algorithm in a specific case.

In the qubit system, the above argument is applied to the mixed states because the Bloch ball is regarded as the hypersphere $S_{+}^{3}$, a hemisphere of a 3-sphere, in a real four-dimensional Euclidean space (e.g., see Section 9.5 in Bengtsson and Życzkowki [29]).

Though the algorithm itself is not our main concern, we briefly mention the efficiency of the algorithm. The computational complexity of each Step (1), (2), and (3) is, respectively, $O(m^{2})$, $O(m^{3})$, and $O(m^{4})$, and clearly it is not efficient. The above problem is reduced to finding the covering sphere for all demand points with the minimum radius (cf. Shiode [19]). Based on this idea, it could be possible to obtain more efficient algorithms even for a continuous model.

The facility location problem on the sphere and finding the representative quantum state of a model (in a two-dimensional Hilbert space) are completely different. It is a bit surprising that the former problem, which comes from operations research, is helpful for understanding the result in the latter, which comes from quantum physics. What a top manager in a global business really cares about might be essentially the same as a fundamental problem in quantum physics. How does this wonderful connection arise? A mathematician might point out the underlying isomorphism between SU(2) and SO(3) [29]. However, this connection arises mainly from a game-theoretic approach. In other words, the unexpected tie implies the universality and effectiveness of game-theoretic concepts, which are different from information-theoretic concepts such as entropy. This is again emphasized when we introduce the definition of model information in the next section.

## 5. Quantum Detection Game and Model Information

We have explained how to determine a representative quantum state of a given model. Based on the state, in the present section, we define a new information quantity, model information. Geometrically, this is the maximum radius from the representative quantum state as the center.

The basic strategy to define an information quantity is to introduce a certain imaginary two-person game where one player obtains points according to the information available.

For example, in classical information theory, we consider assigning the ideal code length −logp(x) to each alphabet *x* when we know the code distribution p(x). Bob’ s best score is given by his guessed distribution q(x) and obtains the score {−logq(x)}−{−logp(x)} for each alphabet *x*. Taking the average with respect to p(x), we obtain the Kullback–Leibler information [23], which is a very fundamental quantity in information theory.

According to Tanaka [20], we consider a quantum detection game as an imaginary two-person game.

### 5.1. Quantum Detection Game and Definition of Purely Quantum Information

As an example, we introduce a four-dimensional pure state model, $M_{FP}$ and set ϵ between 1/4<ϵ<1. This consists of the following four-dimensional vectors:

(11) $$M_{FP}=\left\{\varphi_{1},\varphi_{2},\varphi_{3},\varphi_{4}\right\}$$

(12) $$\varphi_{1}=e_{1}=\left(\begin{array}{c} 1\\ 0\\ 0\\ 0 \end{array}\right),\varphi_{2}=\left(\begin{array}{c} \sqrt{\epsilon }\\ \sqrt{1-\epsilon }\\ 0\\ 0 \end{array}\right),\varphi_{3}=\left(\begin{array}{c} \sqrt{\epsilon }\\ 0\\ \sqrt{1-\epsilon }\\ 0 \end{array}\right),\varphi_{4}=\left(\begin{array}{c} \sqrt{\epsilon }\\ 0\\ 0\\ \sqrt{1-\epsilon } \end{array}\right).$$

while dimH=4, it is enough to consider each vector in a *real* four-dimensional vector space.

Let us explain the quantum detection game under the model $M_{FP}$. First, Alice picks one pure state from the model (i.e., $\varphi_{1},\ldots ,\varphi_{4}$) and then sends it to Bob. Bob knows only the candidate pure state sets and the model, and prepares a two-outcome measurement in the form {|φ〉〈φ|,I−|φ〉〈φ|}, where *I* is the identity operator and φ is the unit vector. We call φ a *detector* or a *detector state*. Bob’s purpose is not to guess the number that Alice has chosen but to obtain "detection" with a higher probability.

The detection rate for the chosen state $|\varphi_{j}\rangle \langle \varphi_{j}|$ is given by $\mathrm{Tr}\{|\varphi_{j}\rangle \langle \varphi_{j}||\varphi \rangle \langle \varphi |\}={\left|\langle \varphi |\varphi_{j}\rangle \right|}^{2}$ when Alice sends $\varphi_{j}$ to Bob and Bob prepares φ as a detector. (Tr denotes the matrix trace and |a〉〈b| is regarded as a matrix). As a game, Alice aims at making Bob’s detection rate smaller by choosing $\varphi_{1},\ldots ,\varphi_{4}$ with a certain probability. In contrast, Bob aims at making the detection rate larger by preparing his detector φ *based on the knowledge of the model*. Later, we will evaluate the information of the model $M_{FP}$.

Now we go back to the general situation and explain the details. First, we seek the minimum detection rate for Bob among all possible models. Suppose that Alice picks among the whole pure states in a completely random way (i.e., with respect to the Haar measure). This is the worst case for Bob. When Bob is allowed to adopt a randomized strategy, the detection rate is 1/d (*d* is the dimension of the Hilbert space). If the model consists of the orthonormal basis, then again the detection rate is 1/d. It is the minimum detection rate.

Next, suppose that Alice has a certain tendency for choosing the pure state, which is also described by the model M and Bob knows this for some reason. Although we do not care about the origin of such models, there are various situations where they apply in quantum science and technology. For example, in the bipartite system $C^{2}⊗C^{2}$ without interaction, a pure state arises as a product state like $\left|\varphi \rangle \right|\varphi^{\prime }\rangle$. Then an entangled state such as α|00〉+β|11〉 is not expected. In quantum computation, the output qubit state under the unitary gate, which has some rotation error, would be $e^{i(\epsilon +\pi /2)Y}|\varphi \rangle$. Then, Bob could obtain a detection rate larger than 1/d based on the information of the model. Following this idea, we propose one information quantity for a model below.

A detailed explanation of the quantum detection game and useful results are described in the author’s previous work [20]. Below, we only present some of the results in a formal way, which is necessary to define the information quantity. Those definitions hold in an infinite-dimensional Hilbert space.

First, we define the Bayes mixture in a slightly formal way. Let $M=\{\psi_{\theta }:\;\theta \in \Theta \}$ be a model, (see Section 3) and π be a probability distribution on the parameter space Θ. Then, the Bayes mixture $\rho_{\pi }$ is defined as

(13) $$\begin{array}{c} \rho_{\pi }=\int_{\Theta }|\psi_{\theta }\rangle \langle \psi_{\theta }|\pi \left(d\theta \right), \end{array}$$

In the context of Bayesian statistics [31,32,33], we call π a *prior distribution* or briefly a *prior*. For a discrete model, the above integral is replaced with a finite sum $\sum_{j}\pi_{j}|\psi_{j}\rangle \langle \psi_{j}|$. Then, when Alice sends $|\psi_{j}\rangle$ to Bob with probability $\pi_{j}$, it is equivalent to sending $\rho_{\pi }$ to Bob in the quantum detection game.

Finally, we have come to our main theme: to define the information quantity of a model M.

**Definition** **2.** *Let M be a model in a d-dimensional Hilbert space. Then, we define the purely quantum information (PQI) of a model M as*

$$\begin{array}{c} J\left(M\right)=\underset{\pi }{\inf}{∥\rho_{\pi }∥}_{\infty }-1/d. \end{array}$$

For calibration, we subtract the lower bound 1/d, and thus J(M)≥0. When Bob knows that the quantum state Alice prepares is among M, we interpret this as Bob obtaining J(M). As shown in Section 5.3, the above infimum is related to the value of the quantum detection game (possible maximum score) through the minimax theorem [20].

Let us rewrite J(M) in a slightly simpler form. For a discrete model, there exists a prior distribution that achieves the infimum of ${∥\rho_{\pi }∥}_{\infty }$. Then, we call the prior a *least favorable prior* (LFP). LFP is one of the technical terms in statistical decision theory or game theory (see, e.g., Section 1.7, p. 39 in Ferguson [30]). Using the least favorable prior $\pi_{LF}$, PQI is defined by

(14) $$\begin{array}{c} J\left(M\right)={∥\rho_{\pi_{LF}}∥}_{\infty }-1/d. \end{array}$$

Even if LFP is not uniquely determined, ${∥\rho_{\pi_{LF}}∥}_{\infty }$ remains the same [20]. As some readers may recognize, the LFP completely agrees with the least favorable weight in Section 4.

### 5.2. Basic Properties of PQI

From the form (14), we obtain some properties of PQI easily. First, by definition, J(M) is independent of a basis. In other words, both $M=\{\psi_{j}\}$ and $M^{\prime }=\left\{U\psi_{j}\right\}$, where *U* is a unitary operator, yield the same PQI. Second, clearly the following holds.

**Lemma** **4.** *Let M be a model in a d-dimensional Hilbert space. The following conditions are equivalent.*

*(i)*  *J(M)=0.*
*(ii)*  *$\rho_{\pi_{LF}}=\frac{1}{d}I$ for every LFP.*

When J(M)=0, Alice can send the completely mixed state effectively and then Bob obtains no information from the model M to achieve a higher detection rate than 1/d. Geometrically speaking, such a model fully spreads with no specific direction.

In contrast, when J(M)>0, a certain bias exists and it prevents Alice from preparing the completely mixed state. Thus, Bob benefits after knowing the model. If M satisfies the full-rank condition, then there exists a prior π such that the Bayes mixture $\rho_{\pi }>0$ (A>0 denotes the positive definiteness of a Hermitian matrix *A*). If M does not satisfy the full-rank condition, we have a $d^{\prime }$-dimensional subspace where M (restricted to the subspace) satisfies the full-rank condition. Since $\inf_{\pi }{∥\rho_{\pi }∥}_{\infty }\geq 1/d^{\prime }>1/d$, we have the lower bound of PQI, $J\left(M\right)\geq 1/d^{\prime }-1/d$.

We mention the relation between PQI and the von Neumann entropy S(ρ). (Recall that the von Neumann entropy is defined by S(ρ)=−Trρlogρ). It is easily shown that J(M)=0 if and only if $S(\rho_{\pi_{LF}})=\log d$. The worst case for Bob also corresponds to the maximum entropy state. As we shall see in Section 6, our formulation is instead related to the minimum entropy.

Next, we consider how to calculate the PQI of a given model. If the model has a certain symmetry, then we obtain the LFP analytically and directly calculate ${∥\rho_{\pi_{LF}}∥}_{\infty }$. On the other hand, due to the minimax theorem in the author’s previous work [20], the infimum of the operator norm of a Bayes mixture, $\inf_{\pi }{∥\rho_{\pi }∥}_{\infty }$ is easily calculated by finding the representative quantum state of the model, which is defined in Section 3. Thus, we are able to calculate the PQI of a given model by finding the minimax point (the representative quantum state of the model), and to do so, we utilize the algorithm shown in Section 4 in order to find the minimax point in the facility location problem on the unit sphere. We present the above procedure explicitly in Section 5.4 in detail.

The mathematical structure is quite similar to the calculation of channel capacity in classical information theory [34,35]. However, we emphasize that even in a formal analogue, we do not introduce any entropic quantity or any concept from information theory to define the above PQI. What we have used is an imaginary two-person game called the quantum detection game and some basic rules in quantum physics. Taking into account many works in quantum information theory [36], it is a bit surprising to develop purely quantum information *without referring to any classical concepts in information theory* [37,38,39,40].

We also note that PQI is completely different from other kind of information quantity such as the Fisher information [7,8]. For a parametric model of quantum states, ρ(θ), differentiable with respect to the parameter θ, Fisher information evaluates the change of quantum states, ρ(θ+Δθ)−ρ(θ). It is related to the distinguishability between two close quantum states ρ(θ) and ρ(θ+Δθ) from observation *after* performing some measurements. Let us take a specific example to see the difference. Suppose that we have a continuous one-parameter model $M_{rot}=\{|\varphi \left(s\right)\rangle =\left(\cos s\right)|0\rangle +\left(\sin s\right)|1\rangle :\;0\leq s\leq \pi /4\}$. Although quantum Fisher information has been defined in various ways as an extension of classical Fisher information, it is not defined for a discrete model such as $M_{2}=\{|0\rangle ,|+\rangle \}$. Indeed, for $M_{2}$, we only consider distinguishing between two possible states (quantum state discrimination), while for $M_{rot}$, we have to consider parameter estimation (quantum state estimation), and the estimation error is bounded by SLD Fisher information [4,5,6]. However, PQI yields the same value for both $M_{2}$ and $M_{rot}$.

### 5.3. Basic Formula for PQI Calculation

We provide several examples to show how we calculate the PQI of a model below. We give the following formula, which connects the representative quantum state and PQI. The formula is obtained by the minimax theorem (10).

(15) $$\begin{array}{cc} 1-{∥\rho_{\pi_{LF}}∥}_{\infty } & =\min_{\varphi }\max_{\psi \in M}d_{F}\left(\varphi ,\psi \right)\\ & =\max_{\psi \in M}d_{F}\left(\varphi_{rep},\psi \right). \end{array}$$

Or equivalently, we have the formula

(16) $$\begin{array}{cc} {∥\rho_{\pi_{LF}}∥}_{\infty } & =\max_{\psi \in M}{\left|\langle \varphi_{rep}|\psi \rangle \right|}^{2}. \end{array}$$

Using the above formula and result in Section 4, we obtain the PQI of $M_{2}$, $M_{3}$, and $M_{4}$ (For the definition, see Section 2.1).

First, we consider the PQI of $M_{2}$. We already know the representative quantum state of $M_{2}$ from Section 4. Thus, using the Formula (16), we obtain ${∥\rho_{\pi_{LF}}∥}_{\infty }={\left|\langle \varphi_{rep}|0\rangle \right|}^{2}=\frac{2+\sqrt{2}}{4}$ and $J\left(M_{2}\right)=\frac{\sqrt{2}}{4}$. In the same way, for $M_{3}$, using the Formula (16), we obtain ${∥\rho_{\pi_{LF}}∥}_{\infty }=\frac{3+\sqrt{3}}{6}$, $J\left(M_{3}\right)=\frac{\sqrt{3}}{6}$. Straightforward calculation also yields $J\left(M_{4}\right)=\frac{\sqrt{3}}{6}$, which coincidently is equal to $J(M_{3})$.

### 5.4. Example of PQI Calculation: $M_{FP}$

Next, as a more nontrivial example, we calculate the PQI of the model $M_{FP}$ introduced in Section 5.1. First, following the algorithm in Section 4, let us find the minimax point (the representative quantum state of the model $M_{FP}$).

In Step (1), we find the most distant pair. We mainly focus on the inner product between two vectors instead of the geodesic distance between them. Then, the most distant pair corresponds to those for which the inner product is the closest to zero. Since $|\langle \varphi_{1}|\varphi_{j}\rangle {|}^{2}=\epsilon$, $|\langle \varphi_{j}|\varphi_{k}\rangle {|}^{2}=\epsilon^{2}$, (j,k=2,3,4), and $0<\epsilon^{2}<\epsilon <1$, the most distant pair is $\left\{\varphi_{2},\varphi_{3}\right\}$, $\left\{\varphi_{2},\varphi_{4}\right\}$, and $\left\{\varphi_{3},\varphi_{4}\right\}$.

Using the Formula (7) in Lemma 2, we obtain the geodesic midpoint between $\varphi_{2}$ and $\varphi_{3}$, which is denoted by $M_{1}$ and $\varphi_{M_{1}}=\frac{1}{\sqrt{2(1+\epsilon )}}{\left(2\sqrt{\epsilon },\sqrt{1-\epsilon },\sqrt{1-\epsilon },0\right)}^{⊤}$. Comparing the inner products, it is easily seen that $\varphi_{1}$ is located at a point more distant from $\varphi_{M_{1}}$ than $\varphi_{2}$ and $\varphi_{3}$. Thus, we go to Step (2) in the algorithm. Note that in the model $M_{FP}$, all inner products are real and positive.

In Step (2), we find the most distant triplet. In our model, it is enough to consider the circumscribed hypercircle in a *real* four-dimensional Euclidean space. Due to the symmetry, we only check two triangles, $\Delta_{234}$ and $\Delta_{123}$ whose vertices are $\left\{\varphi_{2},\varphi_{3},\varphi_{4}\right\}$ and $\left\{\varphi_{1},\varphi_{2},\varphi_{3}\right\}$, respectively.

First, let *Q* be the center of the circumscribed hypercircle of the triangle $\Delta_{234}$. (Each edge is a geodesic on the sphere). Generally, the point *Q* is not uniquely determined. However, by imposing the condition that *Q* is on the three-dimensional real subspace $L_{234}={\mathrm{span}}_{R}\left\{\varphi_{2},\varphi_{3},\varphi_{4}\right\}$, the point *Q* is uniquely determined as the point achieving the minimum distance from each vertex (radius of the circumscribed hypercircle). The condition is equivalent to an orthogonality condition, i.e., $\psi \in L_{234}⟺\langle \psi |\varphi_{L}\rangle =0$, where $|\varphi_{L}\rangle ={\left(\sqrt{1-\epsilon },-\sqrt{\epsilon },-\sqrt{\epsilon },-\sqrt{\epsilon }\right)}^{⊤}$.

Now let $\varphi_{Q}={\left(x,y,z,w\right)}^{⊤}$, x,y,z,w>0 be a vector corresponding to *Q*. Then it satisfies $\langle \varphi_{Q}|\varphi_{2}\rangle =\langle \varphi_{Q}|\varphi_{3}\rangle =\langle \varphi_{Q}|\varphi_{4}\rangle$, $\langle \varphi_{Q}|\varphi_{L}\rangle =0$, and ${∥\varphi_{Q}∥}_{2}^{2}=1$, where ${∥\cdot ∥}_{2}$ denotes the Euclid norm. We obtain the solution $\varphi_{Q}=\frac{1}{\sqrt{3(2\epsilon +1)}}{\left(3\sqrt{\epsilon },\sqrt{1-\epsilon },\sqrt{1-\epsilon },\sqrt{1-\epsilon }\right)}^{⊤}$.

Next, we investigate the other circumscribed hypercircle of the triangle $\Delta_{123}$. In a similar way, we define the point *R* for $\Delta_{123}$. Then, the state vector corresponding to *R* is given by $\varphi_{R}={\left(\sqrt{\frac{1+\sqrt{\epsilon }}{3-\sqrt{\epsilon }}},\sqrt{\frac{1-\sqrt{\epsilon }}{3-\sqrt{\epsilon }}},\sqrt{\frac{1-\sqrt{\epsilon }}{3-\sqrt{\epsilon }}},0\right)}^{⊤}$.

Let each radius of the circumscribed hypercircle be $r_{123}$ and $r_{234}$. Then $r_{123}^{2}=d_{F}\left(\varphi_{R},\varphi_{1}\right)=\frac{2(1-\sqrt{\epsilon })}{3-\sqrt{\epsilon }}$, and $r_{234}^{2}=d_{F}\left(\varphi_{Q},\varphi_{2}\right)=\frac{2(1-\epsilon )}{3}$. Thus, $r_{234}^{2}>r_{123}^{2}$, the most distant triplet is $\left\{\varphi_{2},\varphi_{3},\varphi_{4}\right\}$.

Finally, we check whether the circumscribed hypercircle with center $\varphi_{Q}$ and radius $r_{234}$ includes the other point $\varphi_{1}$. (If not, we go to Step (3) in the algorithm) Since we assume that (1>)ϵ>1/4, $\langle \varphi_{1}|\varphi_{Q}\rangle =\sqrt{\frac{3\epsilon }{1+2\epsilon }}>\langle \varphi_{2}|\varphi_{Q}\rangle =\langle \varphi_{3}|\varphi_{Q}\rangle =\langle \varphi_{4}|\varphi_{Q}\rangle =\sqrt{\frac{1+2\epsilon }{3}}$ holds, this implies that $\varphi_{1}$ is closer to the point $\varphi_{Q}$ than the other three points. Thus, the Algorithm stops and $\varphi_{Q}$ is the minimax location.

Using $\varphi_{Q}$, we obtain

$$\begin{array}{cc} V_{L} & :=\underset{\psi ,{∥\psi ∥}_{2}=1}{\sup}\underset{1\leq j\leq 4}{\inf}{\left|\langle \varphi_{j}|\psi \rangle \right|}^{2}\\ & =\frac{1+2\epsilon }{3}. \end{array}$$

Due to Equation (16), $V_{L}$ agrees with the infimum of the detection rate ${∥\rho_{\pi_{LF}}∥}_{\infty }$, and we obtain PQI $J\left(M_{FP}\right)=\frac{1+8\epsilon }{12}$.

### 5.5. PQI Calculation from LFP

Now let us find the LFP in this model. Since the model $M_{FP}$ has a certain symmetry, we obtain it directly.

First, let the support of π be $\left\{\varphi_{2},\varphi_{3},\varphi_{4}\right\}$, that is, $\pi_{2}+\pi_{3}+\pi_{4}=1$ and $\pi_{1}=0$, $\pi_{j}>0,\;j=2,3,4$. Then we obtain one of the LFPs, which is given by the uniform distribution, $\pi_{2}=\pi_{3}=\pi_{4}=1/3$. To see this, we use the following two facts. First, for every permutation σ (e.g., σ(2)=4,σ(3)=3,σ(4)=2),

$$\begin{array}{cc} {∥\sum_{j=2}^{4}\pi_{\sigma (j)}|\varphi_{j}\rangle \langle \varphi_{j}|∥}_{\infty } & ={∥\sum_{j=2}^{4}\pi_{j}|\varphi_{j}\rangle \langle \varphi_{j}|∥}_{\infty } \end{array}$$

holds. (To see this, construct the unitary operator $U_{\sigma }$, which is the group homomorphism $\sigma \mapsto U_{\sigma }$). Second, the average for every permutation is given by

$$\begin{array}{cc} \frac{1}{3!}\sum_{\sigma \in S_{234}}\sum_{j}\pi_{\sigma (j)}|\varphi_{j}\rangle \langle \varphi_{j}| & =\frac{1}{3}\sum_{j}|\varphi_{j}\rangle \langle \varphi_{j}|, \end{array}$$

where $S_{234}$ is the permutation group acting on the set {2,3,4}. Thus, for every π,

$$\begin{array}{cc} {∥\frac{1}{3}\sum_{j}|\varphi_{j}\rangle \langle \varphi_{j}|∥}_{\infty } & ={∥\frac{1}{3!}\sum_{\sigma \in S_{234}}\sum_{j}\pi_{\sigma (j)}|\varphi_{j}\rangle \langle \varphi_{j}|∥}_{\infty }\\ & \leq \frac{1}{3!}\sum_{\sigma \in S_{234}}{∥\sum_{j}\pi_{\sigma (j)}|\varphi_{j}\rangle \langle \varphi_{j}|∥}_{\infty }\\ & ={∥\sum_{j}\pi_{j}|\varphi_{j}\rangle \langle \varphi_{j}|∥}_{\infty } \end{array}$$

holds. Thus, the uniform distribution is the LFP when $\pi_{1}=0$.

Then, we relax the condition $\pi_{1}=0$. We set the uniformly mixed state as

(17) $$\begin{array}{c} \rho_{\pi_{*}}=\frac{1}{3}|\varphi_{2}\rangle \langle \varphi_{2}|+\frac{1}{3}|\varphi_{3}\rangle \langle \varphi_{3}|+\frac{1}{3}|\varphi_{4}\rangle \langle \varphi_{4}|. \end{array}$$

Then, the operator norm is given by

(18) $$\begin{array}{cc} {∥\rho_{\pi_{*}}∥}_{\infty } & =\frac{1+2\epsilon }{3}. \end{array}$$

From direct but very tedious calculation, we can see that the above achieves the infimum even if we permit $\pi_{1}>0$. Setting $\rho_{\pi }:=\sum_{j=1}^{4}\pi_{j}|\varphi_{j}\rangle \langle \varphi_{j}|$, we obtain

$$\begin{array}{cc} \underset{\pi }{\inf}{∥\rho_{\pi }∥}_{\infty } & =\underset{0\leq p\leq 1}{\inf}{∥p\rho_{\pi_{*}}+\left(1-p\right)|\varphi_{1}\rangle \langle \varphi_{1}|∥}_{\infty }\\ & =\frac{1+2\epsilon }{3}. \end{array}$$

Therefore, we obtain the LFP as

(19) $$\begin{array}{cc} \pi_{*,1} & =0,\;\pi_{*,2}=\pi_{*,3}=\pi_{*,4}=1/3. \end{array}$$

Even when we do not find the representative quantum state directly, we can construct it from the LFP in the following way. Since the Bayes mixture with respect to LFP is given by Equation (17), we find the first eigenvector $\psi_{\pi_{*}}$ with the maximum eigenvalue $\rho_{\pi_{*}}$ (no degeneracy), which is given by $\psi_{\pi_{*}}={\left(\sqrt{\frac{3\epsilon }{2\epsilon +1}},\sqrt{\frac{1-\epsilon }{3(2\epsilon +1)}},\sqrt{\frac{1-\epsilon }{3(2\epsilon +1)}},\sqrt{\frac{1-\epsilon }{3(2\epsilon +1)}}\right)}^{⊤}$. Actually, this agrees with the representative quantum state, $\varphi_{Q}$, in Section 5.4.

Through the minimax theorem [20], we can also directly show that the norm (18) achieves the minimum. To see this, we introduce the following inequality:

$$\begin{array}{cc} V_{U} & :=\underset{\pi }{\inf}\underset{\psi ,{∥\psi ∥}_{2}=1}{\sup}\sum_{j=1}^{4}\pi_{j}{\left|\langle \varphi_{j}|\psi \rangle \right|}^{2}\\ & =\underset{\pi }{\inf}{∥\rho_{\pi }∥}_{\infty }\\ & \leq \frac{1+2\epsilon }{3}. \end{array}$$

Since $V_{U}\geq V_{L}$ (for details, see the author’s previous work [20]), we obtain $\frac{1+2\epsilon }{3}\geq V_{U}\geq V_{L}=\frac{1+2\epsilon }{3}$, which implies $V_{U}=V_{L}$. Thus, one LFP is given by (19). The above argument does not exclude another possibility for the LFP with $\pi_{1}>0$.

### 5.6. Difference from Maximization of von Neumann Entropy

In our formulation, PQI has no direct relation to any entropic concept. Since some readers may expect a certain relationship, let us see what happens if we formally adopt the von Neumann entropy to obtain the LFP in the last example. We consider the maximization of $S(\rho_{\pi })$ over the prior π. The concavity of S(ρ) yields

$$\begin{array}{cc} \max_{\pi }S\left(\rho_{\pi }\right) & =\max_{0\leq \lambda \leq 1}S(\lambda |\varphi_{1}\rangle \langle \varphi_{1}|+\left(1-\lambda \right)\rho_{\pi_{*}})\\ & \geq \max_{0\leq \lambda \leq 1}\left\{\lambda S(|\varphi_{1}\rangle \langle \varphi_{1}|)+\left(1-\lambda \right)S\left(\rho_{\pi_{*}}\right)\right\}\\ & =S(\rho_{\pi_{*}}), \end{array}$$

where $\pi_{*}$ denotes LFP (19). For some ϵ, we numerically find a positive $\lambda_{*}$ achieving the maximum of $S\left(\lambda |\varphi_{1}\rangle \langle \varphi_{1}|+\left(1-\lambda \right)\rho_{\pi_{*}}\right)$. Thus, a positive weight for $|\varphi_{1}\rangle \langle \varphi_{1}|$ could appear under the maximization, which is clearly different from our result.

While the LFP $\pi_{*}$, which is obtained by minimization of ${∥\rho_{\pi }∥}_{\infty }$, yields the minimax solution in the quantum detection game, the maximizer, say, $\pi_{ent}$, is meaningless, at least in this example. Indeed, ${∥\rho_{\pi_{ent}}∥}_{\infty }>{∥\rho_{\pi_{*}}∥}_{\infty }$, which implies that $\rho_{\pi_{ent}}$ is more informative than $\rho_{\pi_{*}}$, and the prior $\pi_{ent}$ is not the least favorable to Bob anymore.

We have carefully treated the information or uncertainty of a nonorthogonal pure state model and excluded classical fluctuation. As a consequence, the remaining uncertainty is not evaluated by the usual entropy anymore. For a nonorthogonal pure state model, the von Neumann entropy as a measure of information lacks theoretical justification. At least in the quantum detection game, the method based on the von Neumann entropy is a mere formal extension. It makes sense only for a model that consists of orthogonal pure states (see Section 2).

However, there are many variants of entropy [37,38,39,40,41] both in classical and quantum information theory. We discuss a certain relationship between our information quantity and the minimum entropy in the next section.

## 6. Discussion: Relation to Entropy

In the previous section, we introduced an information quantity for a pure state model called PQI. Under the full-rank condition, any classical model consists of an orthonormal basis. Then PQI of the model necessarily vanishes.

We emphasize that PQI is literally something *purely quantum* since we have not formally extended something in classical information theory. It is the information quantity completely independent of the concept of entropy, which does make sense in classical information theory. Thus, a natural question arises, i.e., what kind of relationship do the entropy and PQI have? Actually, PQI is related to the minimum entropy instead of the von Neumann entropy, as we will discuss below.

### 6.1. Jaynes Principle and Distinguishability

First, we briefly review the concept of entropy and the Jaynes principle [42,43].

Suppose that we are given the set of the alphabet. Then our lack of knowledge on the set is evaluated by Shannon entropy through a probability distribution $\left\{p_{i}\right\}$ satisfying $p_{1}+\ldots +p_{d}=1,\;p_{i}\geq 0,\;i=1,\ldots ,d$. (Recall that classical Shannon entropy is defined as $S_{cl}\left(p\right)=\sum_{i}-p_{i}\log p_{i}$). The larger the entropy becomes, the larger the uncertainty we have.

We have minimum information as interpreted as the maximum entropy state, that is, $p_{i}=1/d$ and thus

$$\begin{array}{c} \max_{p}S_{cl}\left(p\right)=\log d \end{array}$$

holds. The central idea also provides the theoretical foundation for maximum entropy methods in data processing [44,45].

The underlying concept is distinguishability. In classical information theory, distinguishability holds trivially. In quantum theory, it is represented by the orthogonality of two quantum states. When pure states corresponding to alphabets, say, {1,2,…,d}, are orthogonal to each other, every result in classical information theory is extended in a straightforward manner.

In statistical physics, a physical state of an ensemble is estimated through entropy maximization when we have no knowledge of the system. This way of thinking is called the *Jaynes principle* [42,43], and it is fundamental to statistical physics. For example, for a given set of eigenstates of a Hamiltonian, say, $|E_{0}\rangle ,|E_{1}\rangle ,\ldots$, with some conditions, we obtain a canonical ensemble by using the principle.

In quantum physics, we are able to consider the maximization of the von Neumann entropy of the density matrix $\rho_{\pi }=\sum_{j}\pi_{j}|\psi_{j}\rangle \langle \psi_{j}|$ (Bayes mixture) for orthogonal vectors $\left\{\psi_{1},\ldots ,\psi_{d}\right\}$. Since $S\left(\rho_{\pi }\right)=S_{cl}\left(\pi \right)$, this maximization completely reduces to the classical case. Then the maximizer is the completely mixed state, i.e., $\frac{1}{d}I$, which corresponds to the uniform distribution. Formally, additional constraints also yield a quantum exponential family [46], which is the quantum analogue of the classical exponential family [47,48].

However, we have no solid criterion such as the Jaynes principle for a nonorthogonal pure states model. For example, a qubit |0〉 processed under one unitary operation, which is assumed to be among $U_{1},U_{2},U_{3}$. ($|\psi_{j}\rangle =U_{j}|0\rangle$, j=1,2,3). In a sense, it is a simplified rotation error model (e.g., Kueng et al. [2]). In our formulation, a model $M_{U}=\left\{\psi_{1},\psi_{2},\psi_{3}\right\}$ is given. Suppose that we have no information or knowledge on which unitary gate processed the qubit. Then, how do we describe the quantum bit?

Mathematically, it is possible to extend the maximum entropy criterion to the noncommutative case. Then we consider the maximization,

(20) $$\begin{array}{c} \underset{\pi }{\sup}S\left(\rho_{\pi }\right) \end{array}$$

over the prior π. Is this kind of formal extension enough in quantum information theory? There are many quantities such as Rényi’s entropy [37,38,39,40,41] in both classical and quantum information theory. Is there another possibility to consider such quantities?

Every quantum state in the model $M_{U}$ is not orthogonal anymore; thus, they are not distinguishable, which is completely different from the set of the alphabet. In spite of this, do we seek some justification for the maximization of the entropy from classical information theory?

In our formulation, the above formal argument breaks down. First, for the model $M_{U}$, we describe the system by the representative quantum state $\varphi_{rep}\left(M_{U}\right)$, which is completely independent of the von Neumann entropy. Second, in the quantum detection game between Alice and Bob, we see that the von Neumann entropy is useless in a specific example (Section 5.6). Rather, we consider the least favorable case or the minimization of the detection rate, $\inf_{\pi }{∥\rho_{\pi }∥}_{\infty }$, which is contrastive to the maximization of entropy (20).

If we seek a purely quantum counterpart of the Jaynes principle, then minimization of ${∥\rho_{\pi }∥}_{\infty }$ would be promising. Luckily, due to monotonicity, the minimization is equivalent to the maximization of $-\log{∥\rho_{\pi }∥}_{\infty }$, where the function $-\log{∥\rho ∥}_{\infty }$ is known as the *minimum entropy* of ρ. Some of its properties are similar to those of the von Neumann entropy and others are not. In the next subsection, we review basic properties of the minimum entropy.

### 6.2. Properties of the Minimum Entropy

In the present subsection, we briefly review basic properties of the minimum entropy and then give another definition for purely quantum information. The minimum entropy of the density matrix ρ is defined by $T\left(\rho \right):=-\log{∥\rho ∥}_{\infty }$, which is a special case of quantum Rényi entropy.

Quantum Rényi entropy has a real parameter α and is defined by

$$\begin{array}{cc} S_{\alpha }\left(\rho \right) & =\frac{\log\mathrm{Tr}\rho^{\alpha }}{1-\alpha } \end{array}$$

for fixed α (see, e.g., p. 117 in Ohya and Petz [41]). When α→∞, we obtain the minimum entropy.

The minimum entropy T(ρ) inherits some common properties of quantum Rényi entropy. For example, additivity T(ρ⊗σ)=T(ρ)+T(σ) holds. For every pure state, *T* equals to zero.

However, the concavity does not necessarily hold. Concavity of entropy means that a probability mixture of quantum states increases the uncertainty of the whole system.

This negative property is not due to noncommutativity. To see this, let us take two commutative density matrices,

$$\rho =\left(\begin{array}{cc} 1 & 0\\ 0 & 0 \end{array}\right),\;\sigma =\left(\begin{array}{cc} \mu & 0\\ 0 & 1-\mu \end{array}\right),$$

where $\frac{1}{2}\leq \mu <1$. Then, since ${∥\frac{\rho +\sigma }{2}∥}_{\infty }=\frac{1+\mu }{2}$,

$$\begin{array}{cc} \frac{1}{2}T\left(\rho \right)+\frac{1}{2}T\left(\sigma \right)-T\left(\frac{\rho +\sigma }{2}\right) & =\log\left(\frac{1+\mu }{2\sqrt{\mu }}\right)>0 \end{array}$$

Thus, convexity rather than concavity holds in the above example.

Since minimum entropy is based on the operator norm, we obtain a sufficient condition for convexity easily.

**Lemma** **5.** *Let two density matrices ρ and σ exist where ${∥\rho ∥}_{\infty }\neq {∥\sigma ∥}_{\infty }$. Suppose that*

$$\begin{array}{c} {∥\lambda \rho +(1-\lambda )\sigma ∥}_{\infty }=\lambda {∥\rho ∥}_{\infty }+\left(1-\lambda \right){∥\sigma ∥}_{\infty } \end{array}$$

*holds for every λ, 0<λ<1. Then*

$$\begin{array}{c} T(\lambda \rho +(1-\lambda )\sigma )<\lambda T(\rho )+(1-\lambda )T(\sigma ) \end{array}$$

*holds.*

**Proof.**  Since −log(x) is convex,

$$\begin{array}{cc} T(\lambda \rho +(1-\lambda )\sigma ) & =-\log{∥\lambda \rho +(1-\lambda )\sigma ∥}_{\infty }\\ & =-\log\left\{\lambda {∥\rho ∥}_{\infty }+\left(1-\lambda \right){∥\sigma ∥}_{\infty }\right\}\;∵\mathrm{Assump}.\\ & <\lambda \left(-\log{∥\rho ∥}_{\infty }\right)+\left(1-\lambda \right)\left(-\log{∥\sigma ∥}_{\infty }\right)\\ & =\lambda T(\rho )+(1-\lambda )T(\sigma ) \end{array}$$

holds. □

To see the above lemma, when we introduce T(ρ) as a variant of entropy over the whole density operators, its theoretical significance seems very weak.

However, when we consider PQI in a pure state model, the situation changes drastically. For the pure state family, concavity of the minimum entropy necessarily holds in the following sense.

**Lemma** **6.** *Let a set of pure states be given, say M. Then concavity holds, restricted to the model.*

**Proof.**  Choose a finite set of pure states from M, say $\rho_{1},\ldots ,\rho_{k}$. Then

$$\begin{array}{c} T\left(\sum_{j=1}^{k}\lambda_{j}\rho_{j}\right)\geq 0=\sum_{j=1}^{k}\lambda_{j}T\left(\rho_{j}\right) \end{array}$$

holds. □

Other properties of minimum entropy are usually shown in the context of quantum Rényi entropy (see, e.g., Hu and Ye [22] (Section III, p. 4), Dam and Hayden [21]).

Observing the above, we provide another possible definition of purely quantum information through T(ρ). In the quantum detection game, finding the LFP is equivalent to maximizing the minimum entropy $T(\rho_{\pi })$ rather than the von Neumann entropy $S(\rho_{\pi })$. To consider the logarithm of the detection rate, we obtain another definition of purely quantum information such as

$$\begin{array}{c} J^{\prime }\left(M\right)=\log d-\underset{\pi }{\sup}T\left(\rho_{\pi }\right)\;\left(\geq 0\right). \end{array}$$

By definition, $J^{\prime }\left(M\right)$ vanishes if the model has orthogonal pure states with the full-rank condition, where the classical case is included.

From Lemmas 5 and 6, we must be careful to treat minimum entropy T(ρ) in the definition of $J^{\prime }\left(M\right)$. At least, it should not be considered over the set of the whole density matrices. As a consequence, a comparison of the two definitions, J(M) and $J^{\prime }\left(M\right)$, also should be performed carefully and would require a deeper understanding of the model information, which will be a topic for future research.

Finally, we make two comments. First, our definition of the model information yields one operational meaning for the minimum entropy. It is apart from the usual extension of entropic concepts in classical information theory. Rather, it comes from an imaginary design of the quantum detector and facility location problem on the unit sphere in a complex projective space. Second, we expect that the purely quantum version of the Jaynes principle is established based on the minimum entropy. (For some related works on maximum entropy methods, see the reference [49]). It might be possible to develop data processing methods and some dynamics based on the new principle.

## 7. Infinite-Dimensional Hilbert Space

Thus far, we have considered the PQI of a model only in a finite-dimensional Hilbert space. While our definition of PQI applies to infinite-dimensional Hilbert space, technical difficulties seem to arise due to a parametric family of functions. In this section, we only skim them in a specific example.

Let $L^{2}\left(R\right)$ denote the set of square integrable complex functions over R and *g* be a known continuous function in $L^{2}\left(R\right)$ satisfying ${∥g∥}_{2}^{2}=\int {\left|g\left(x\right)\right|}^{2}dx=1$. Let us consider the quantum statistical model $M_{\infty }=\left\{g\left(x-\theta \right):\theta \in R\right\}$ describing a wavefunction with a single parameter.

Parameter estimation of the shift parameter θ has been theoretically investigated [3]. If we replace the wavefunction *g* with a probability density function such as the Gaussian density, the estimation problem for the shift parameter θ is called that for the location parameter and is very common in classical statistics [50].

Before evaluating the PQI of the model $M_{\infty }$, first let us formally consider quantum state estimation with no observation. It is seen that the worst-case error equals one for every estimation.

**Lemma** **7.** *Let $g_{\theta }\left(x\right)=g\left(x-\theta \right)$ and $f\in L^{2}\left(R\right)$ with ${∥g_{\theta }∥}_{2}={∥f∥}_{2}=1$. Then*

(21) $$\begin{array}{c} \underset{\theta \in R}{\inf}\left|\langle g_{\theta }|f\rangle \right|=0, \end{array}$$

(22) $$\begin{array}{c} \underset{\theta \in R}{\sup}\left\{1-{\left|\langle g_{\theta }|f\rangle \right|}^{2}\right\}=1 \end{array}$$

*holds.*

For proof, see the Appendix A.1. The above lemma says that every quantum state in $L^{2}\left(R\right)$ would be a minimax location on “$CP^{\infty }$”.

Since the parameter space Θ=R is noncompact, the minimax theorem [20] does not hold generally. However, we directly show that the Formula (16) holds in this specific example, that is,

$$\begin{array}{c} \underset{\pi }{\inf}{∥\rho_{\pi }∥}_{\infty }=0=\underset{f\in L^{2}\left(R\right)}{\sup}\left\{\underset{\theta \in R}{\inf}{\left|\langle g_{\theta }|f\rangle \right|}^{2}\right\}. \end{array}$$

The first equality holds due to the following lemma. Because of technical difficulties, we give a proof in the Appendix A.2.

**Lemma** **8.** *Let $g_{\theta }\left(x\right)=g\left(x-\theta \right)$ with ${∥g_{\theta }∥}_{2}=1$ and ϵ be an arbitrary positive constant. Then there exists a finite set $\left\{\theta_{1},\ldots ,\theta_{n}\right\}$ and the uniform prior $\pi_{n}$ over the set such that*

$$\begin{array}{cc} {∥\rho_{\pi_{n}}∥}_{\infty } & \leq \frac{1}{n}+\epsilon ,\\ \rho_{\pi_{n}} & =\frac{1}{n}\sum_{j=1}^{n}|g_{\theta_{j}}\rangle \langle g_{\theta_{j}}|. \end{array}$$

Thus, a formal definition of PQI shows that $J\left(M_{\infty }\right)=\inf_{\pi }{∥\rho_{\pi }∥}_{\infty }=0$. We can interpret the result as follows. Even if Bob knows that the quantum state is in the model $M_{\infty }$ or that the quantum system is described by a wavefunction g(x−θ), he obtains no information, which gives Bob an advantage over Alice in the quantum detection game.

We do not obtain the conditions where PQI is positive with explicit examples. Even if the Formula (16) holds under some conditions, calculation of PQI would become drastically different. A detailed investigation is left for future study.

## 8. Concluding Remarks

We have defined one information quantity called purely quantum information (PQI) not for a pure state itself, but for a parametric model of pure states. While PQI evaluates the size of a pure state model, it necessarily vanishes in classical cases by definition. We call the center of the model the representative quantum state, and PQI is determined by the maximum length from the center to each quantum state in the model.

Finally, we give the answer to the problem presented in the beginning of the article. Let $\psi_{\theta }=e^{-i\theta_{2}Z}e^{-i\theta_{1}Y}|0\rangle$ and calculate PQI for two models:

$$\begin{array}{cc} M_{5} & =\{\psi_{\theta }:\;0\leq \theta_{1}\leq \pi /4,\;0\leq \theta_{2}\leq \pi /4\},\\ M_{6} & =\{\psi_{\theta }:\;0\leq \theta_{1}\leq 2\pi /5,\;\theta_{2}=0\} \end{array}$$

Model $M_{5}$ has the same amount of PQI as $M_{4}$, $J\left(M_{5}\right)=\sqrt{3}/6=0.289$. The PQI of the model $M_{6}$ is $J\left(M_{6}\right)=\frac{1}{2}\cos\frac{2\pi }{5}=0.154$, which is smaller than $J(M_{5})$. This implies that $M_{6}$ spreads more than $M_{5}$, and we see that the PQI is independent of the dimension of the parameter space.

## Data Availability

Not applicable.

## Appendix A. Proofs

### Appendix A.1. Proof of Lemma 7

For ϵ>0, we show that there exists *M* such that

(A1) $$\begin{array}{c} \left|\theta \right|\geq M\Rightarrow \left|\langle f|g_{\theta }\rangle \right|<2\sqrt{\epsilon }. \end{array}$$

First, we take compact sets *K* and *L* satisfying $\int_{K}{\left|f\right|}^{2}dx>1-\epsilon$ and $\int_{L}{\left|g\right|}^{2}dx>1-\epsilon$, respectively. Second, there exists a positive constant *M* such that $\left|\theta \right|\geq M\Rightarrow L+\theta =\left\{x+\theta :\;x\in L\right\}$ and *K* are disjoint. Note that $\int_{L+\theta }{\left|g_{\theta }\right|}^{2}dx>1-\epsilon$ for every θ. Then, we bound the absolute value of the inner product by two terms:

$$\begin{array}{cc} \left|\langle f|g_{\theta }\rangle \right| & \leq \left|\int_{K}\bar{f}\left(x\right)g_{\theta }\left(x\right)dx\right|+\left|\int_{∼K}\bar{f}\left(x\right)g_{\theta }\left(x\right)dx\right|, \end{array}$$

where ∼K denotes the complement of *K*. Due to the Cauchy–Schwarz inequality, the second term is bounded by

$$\begin{array}{cc} \left|\int_{∼K}\bar{f}\left(x\right)g_{\theta }\left(x\right)dx\right| & \leq \sqrt{\int_{∼K}{\left|f\right|}^{2}dx}\sqrt{\int_{∼K}{\left|g_{\theta }\right|}^{2}dx}\\ & \leq \sqrt{\epsilon }{∥g∥}_{2}\\ & =\sqrt{\epsilon }. \end{array}$$

The first term requires more steps. Since K⊂∼(L+θ),

$$\begin{array}{cc} \left|\int_{K}\bar{f}\left(x\right)g_{\theta }\left(x\right)dx\right| & \leq \int_{K}\left|\bar{f}\left(x\right)g_{\theta }\left(x\right)\right|dx\\ & \leq \int_{∼(L+\theta )}\left|\bar{f}\left(x\right)g_{\theta }\left(x\right)\right|dx\\ & \leq \sqrt{\int_{∼(L+\theta )}{\left|f\right|}^{2}dx}\sqrt{\int_{∼(L+\theta )}{\left|g_{\theta }\right|}^{2}dx}\\ & \leq {∥f∥}_{2}\sqrt{\epsilon }\\ & =\sqrt{\epsilon }. \end{array}$$

Putting them together, we obtain (A1). Therefore $|\langle g_{\theta }|f\rangle |\to 0$ as |θ|→0 and we obtain (21) and (22).

### Appendix A.2. Proof of Lemma 8

First from (A1), $|\langle g_{\theta }|g_{\theta^{\prime }}\rangle \left|=\right|\langle g|g_{\theta^{\prime }-\theta }\rangle |\to 0$ when $|\theta -\theta^{\prime }|\to 0$. Thus, for ϵ>0, there exists *M* such that $\left|\theta -\theta^{\prime }\right|\geq M\Rightarrow \left|\langle g_{\theta }|g_{\theta^{\prime }}\rangle \right|<\epsilon$.

Using the above, we construct the sequence of a prior distribution with finite support (i.e., discrete probability) and Bayes mixture. First, for fixed *n*, we take a parameter set $\left\{\theta_{1},\ldots ,\theta_{n}\right\}$ satisfying $|\theta_{i}-\theta_{j}|\geq M$ for i,j=1,…,n.

Without loss of generality, we assume that $g_{\theta_{1}},\ldots ,g_{\theta_{n}}$ are linearly independent. Then, set $G_{ij}=\langle g_{\theta_{i}}|g_{\theta_{j}}\rangle$ ($G_{ii}=1$). It is easily shown that the gram matrix $G=[G_{ij}]$ is positive definite. Then, we decompose *G* into the identity $I_{n}$ and $A=G-I_{n}$, where diagonal components of *A* are zero.

For a uniform weight over the parameter set $\left\{\theta_{1},\ldots ,\theta_{n}\right\}$, we define the Bayes mixture

$$\begin{array}{c} \rho_{n}=\frac{1}{n}\sum_{j=1}^{n}|g_{\theta_{j}}\rangle \langle g_{\theta_{j}}|. \end{array}$$

Now we show that

(A2) $$\begin{array}{cc} {∥\rho_{n}∥}_{\infty } & =\frac{1}{n}{∥G∥}_{\infty }=\frac{1}{n}+\frac{1}{n}{∥A∥}_{\infty }, \end{array}$$

(A3) $$\begin{array}{cc} {∥A∥}_{\infty } & \leq (n-1)\epsilon . \end{array}$$

First, we expand an arbitrary normalized vector ψ as $\psi =\sum_{j}c_{j}|g_{\theta_{j}}\rangle$. Then $1=\langle \psi |\psi \rangle =c^{†}Gc$, where $c={\left(c_{1},\ldots ,c_{n}\right)}^{⊤}$ is a column vector and $c^{†}$ denotes the conjugate transpose of the vector *c*. Since *G* is positive definite, we take another parameter vector *d* as $d=\sqrt{G}c$. Note that there is one-to-one correspondence between ψ and *d*. Thus,

$$\begin{array}{cc} \langle \psi |\rho_{n}|\psi \rangle & =\frac{1}{n}\sum_{j}{\left|\langle \psi |g_{\theta_{j}}\rangle \right|}^{2}\\ & =\frac{1}{n}{∥Gc∥}_{2}^{2}\\ & =\frac{1}{n}d^{†}Gd, \end{array}$$

where ${∥a∥}_{2}^{2}=aa^{†}$.

This implies that

$$\begin{array}{cc} {∥\rho_{n}∥}_{\infty } & =\underset{\psi :{∥\psi ∥}_{2}=1}{\sup}\langle \psi |\rho_{n}|\psi \rangle \\ & =\frac{1}{n}\underset{d:{∥d∥}_{2}=1}{\sup}d^{†}G\;d\\ & =\frac{1}{n}+\frac{1}{n}\underset{d:{∥d∥}_{2}=1}{\sup}d^{†}A\;d\\ & =\frac{1}{n}+\frac{1}{n}{∥A∥}_{\infty }, \end{array}$$

which shows Equation (A2).

Next, we show the inequality (A3). Due to Geršgorin’s Theorem (see, e.g., Section 6.1 in Horn and Johnson [51]), all eigenvalues of *A* are located in the union of *n* discs

$$\begin{array}{c} \overset{n}{\underset{i=1}{⋃}}\left\{z\in C:\;|z|\leq \sum_{j\neq i}\left|G_{ij}\right|\right\}. \end{array}$$

Thus, we easily show that the absolute value of each eigenvalue is bounded by (n−1)ϵ.

Finally, we obtain from (A2) and (A3), ${∥\rho_{n}∥}_{\infty }\leq \frac{1}{n}+\epsilon$. For fixed ϵ>0, we have $0\leq \inf_{\pi }{∥\rho_{\pi }∥}_{\infty }\leq \epsilon$. Since ϵ is arbitrary, $\inf_{\pi }{∥\rho_{\pi }∥}_{\infty }$ must be zero.
